# Late onset axial spondyloarthritis presenting with fever of unknown origin. A case-based review

**DOI:** 10.1007/s00296-026-06079-3

**Published:** 2026-02-20

**Authors:** Paraskevi V. Voulgari, Anastasia K. Zikou, Alexandros A. Drosos

**Affiliations:** 1 Rheumatology Clinic, Department of Internal Medicine, Medical School, University of Ioannina, 45110 Ioannina, Greece; 2 Department of Radiology, Medical School, University of Ioannina, Stavrou Niarchou Blv, 45500 Ioannina, Greece

**Keywords:** Spondyloarthritis, Axial-spondyloarthritis, Fever of unknown origin, Late onset disorders, Acute phase proteins

## Abstract

Spondyloarthritis (SpA) is a chronic inflammatory disease involving axial (ax-SpA), and peripheral joints (p-SpA). It mainly affects young individuals and symptoms begin before the age of 45 years. Late onset (LO) ax-SpA, presenting after the age of 50, is rare. It is usually underdiagnosed in favor of other disorders, which are very common in this population. A 70-year-old woman presented with fever of unknown origin (FUO), back pain, malaise, weakness and high acute phase reactants, without any other clinical or laboratory findings. After an intensive and extensive investigation, to exclude several diseases and disorders, our patient, despite being old and almost asymptomatic for SpA features was diagnosed as having LO-axSpA. The patient was treated successfully with adalimumab. We discuss the differential diagnosis of FUO in an elderly woman with constitutional symptoms and elevated inflammatory markers. This is the first case of LO-axSpA presenting with FUO, therefore, physicians must be aware of rare manifestations of ax-SpA in the elderly population. Rigorous and extensive investigation is required to reach the correct diagnosis.

## Introduction

Spondylarthritis (SpA) is a chronic inflammatory disease affecting axial skeleton (ax-SpA), as well as peripheral joints and entheses (p-SpA). It mainly occurs in young individuals and symptoms of ax-SpA usually begin before the age of 45 [1]. However, “late onset” form of SpA (LO-SpA) has also been infrequently reported in older than fifty years patients [2, 3]. These patients present mostly p-SpA features, without family history of SpA and are less likely to have positive test for human leucocyte antigen (HLA) -27 [4, 5].

All SpA phenotypes such as psoriasis, psoriatic arthritis (PsA), inflammatory bowel disease (IBD) occur in LO-SpA. They are usually undiagnosed in favor of other common inflammatory disorders in this population. Thus, elderly people need careful evaluation because of the presence of various comorbidities and disorders, in order to reach a correct diagnosis [6]. Based on the above, we report a 70-year-old woman who presented with fever of unknown origin (FUO), lasting for 6 months, along with weakness, malaise, back pain and high acute phase reactants. After a meticulous clinical, laboratory and imaging investigation, our patient was diagnosed with LO-axSpA. We also discuss the differential diagnosis of FUO in an elderly woman with constitutional symptoms and elevated acute phase reactants. This study was performed in accordance with the Helsinki Declaration of 1964 and its later amendments. All presented material is published after written consent of the patient, although sensitive data and personal details are not included in the publication.

## Case presentation

**Fig. 1:**
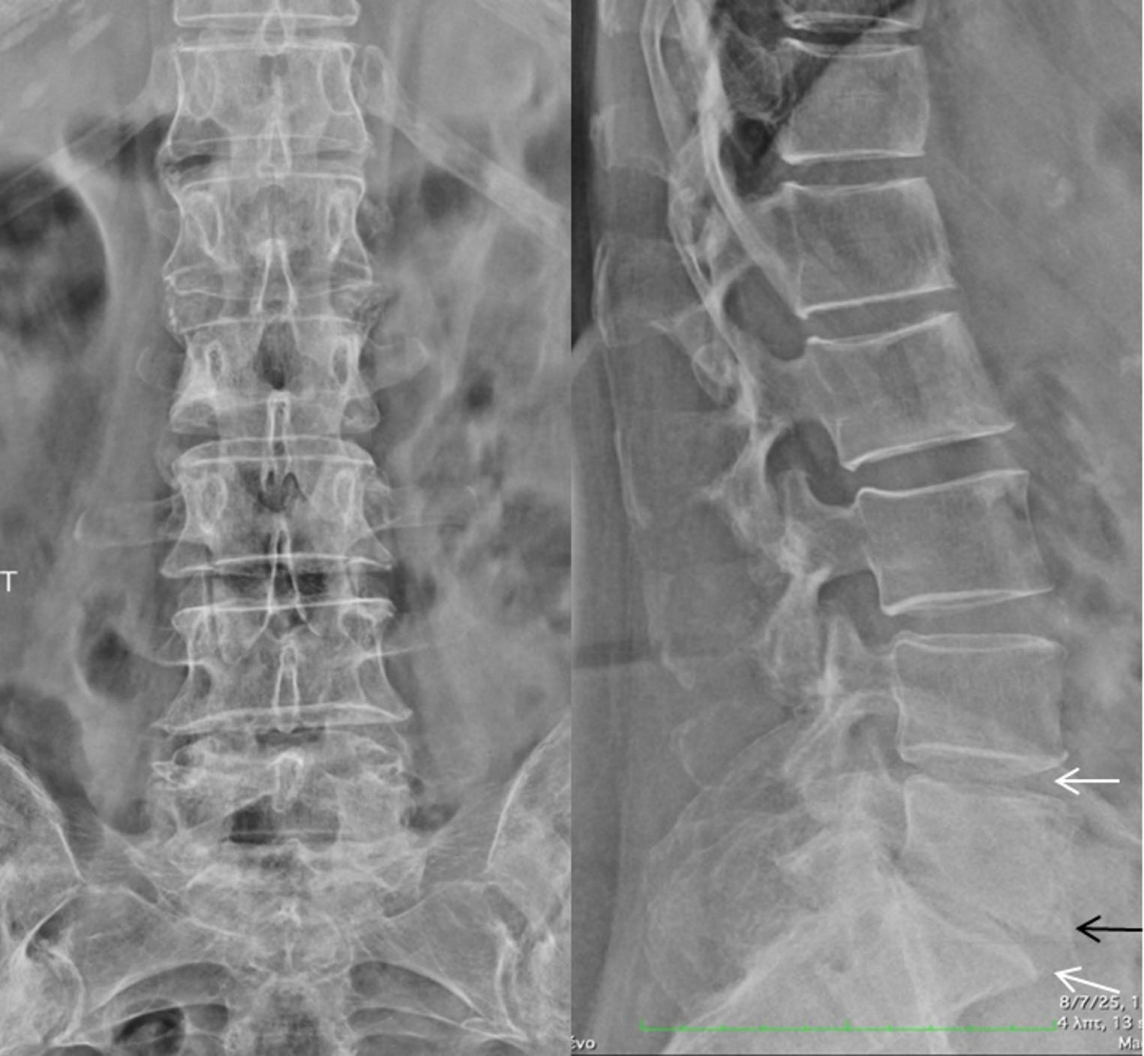
Lumbar spine x-rays, anteroposterior (a) and lateral (b) views. Note the spinal disc space narrowing between the L4-L5 and L5-S1 vertebrae (white arrows) and the presence of osteophytes at the L5-SI level (black arrow).

**Fig. 2:**
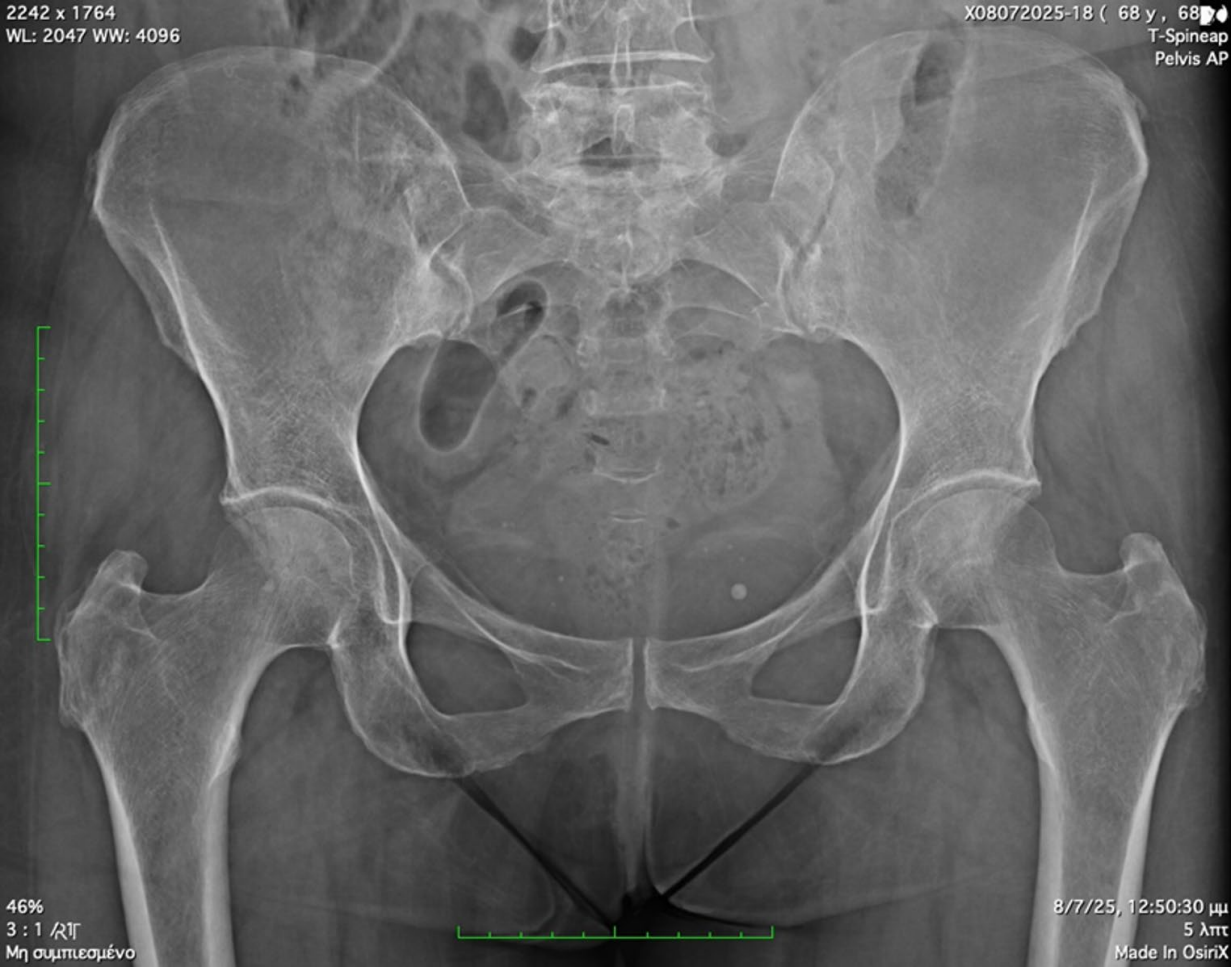
Pelvis x-ray, anteroposterior view. Note that the sacroiliac joints are of uniform width and the cortical lines along joints are intact. The hip joints are also normal.

**Fig. 3:**
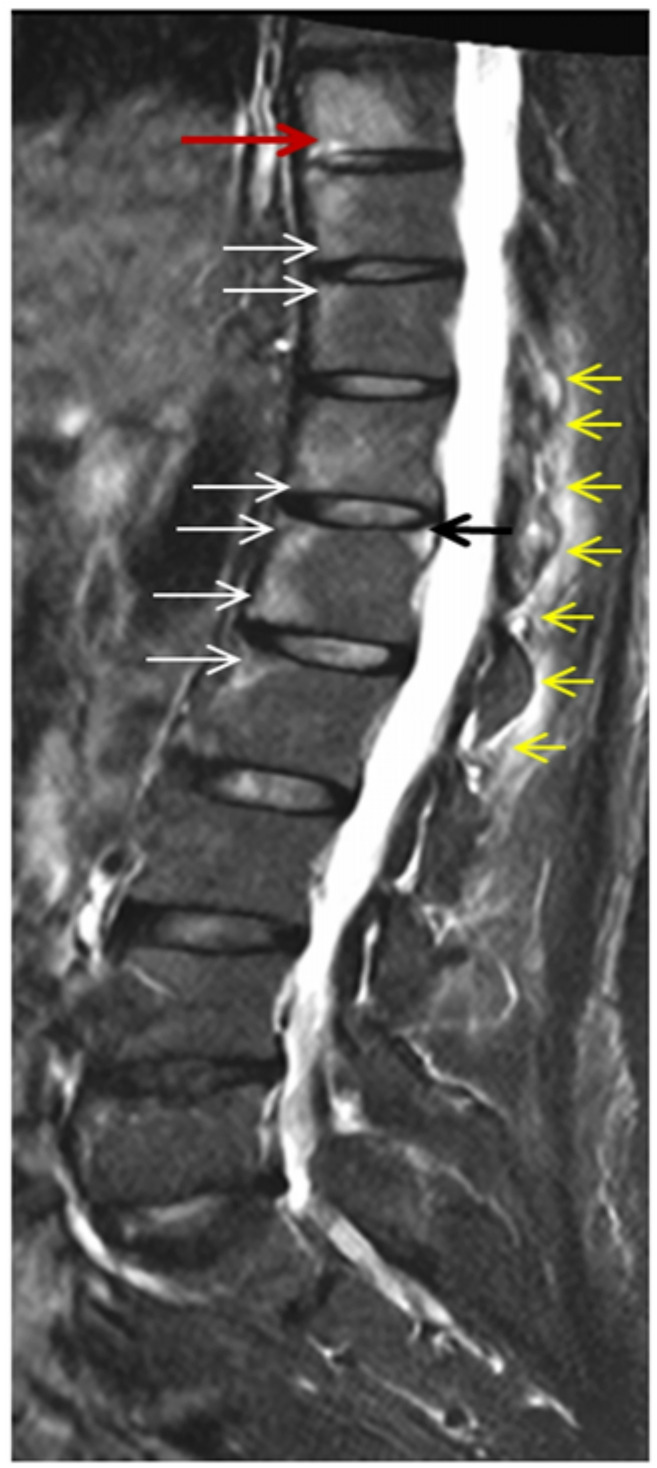
Spinal sagittal MR STIR image shows hyperintense (bone marrow edema) of active Andersson lesion along inferior endplate of T9 (red arrow) and active Romanus lesions within the anterior corner at T10-11, T12-L1, L1-L2 (white arrows) and superior posterior corner at L1 vertebral body (black arrow). Hyperintense active enthesitis at T11-L2 spinous process (yellow arrows).

**Fig. 4:**
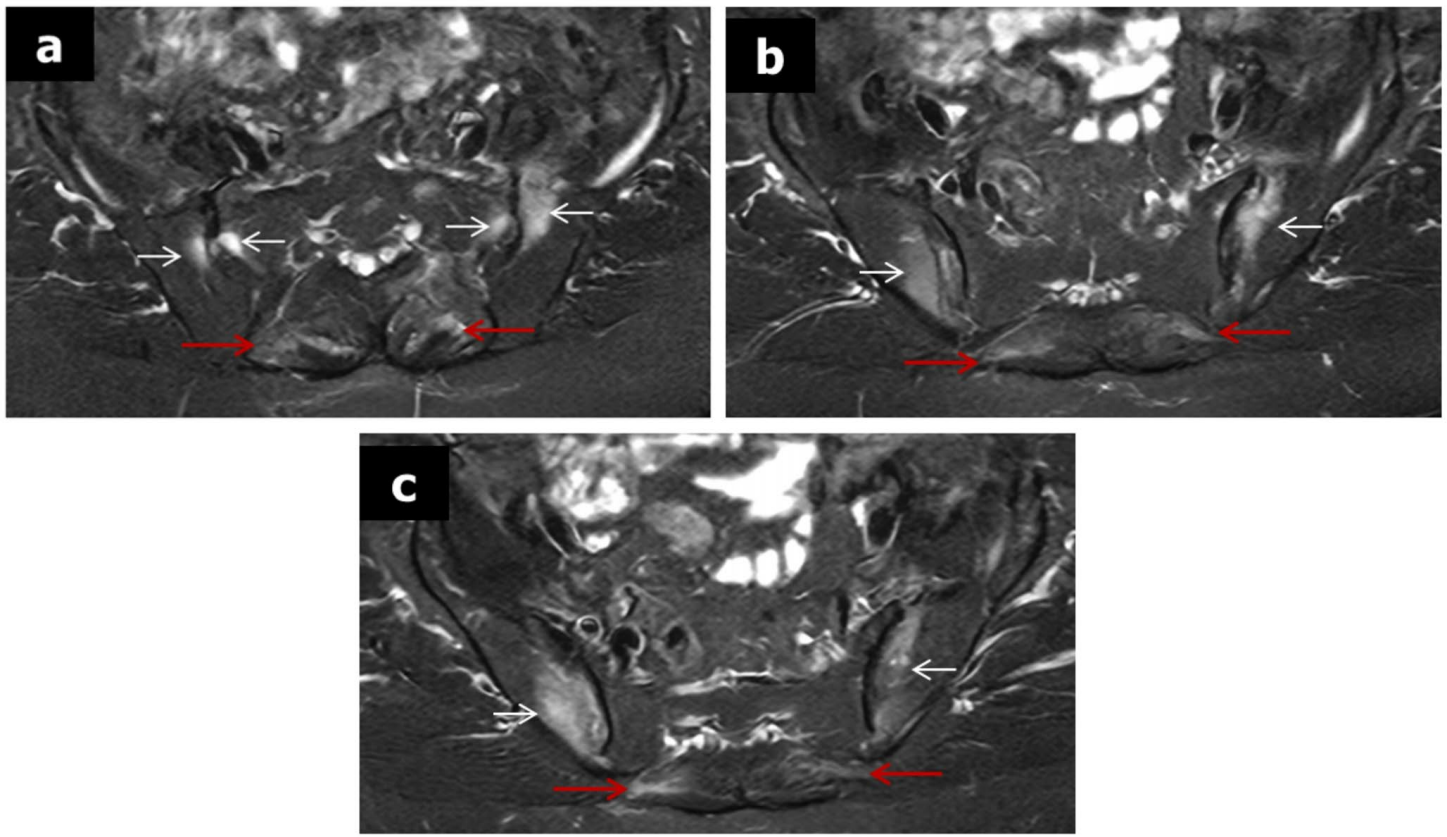
Sacroiliac joints axial MR images (a, b,c) T2-weighted fat suppressed images show bilateral subchondral bone marrow edema (white arrows) and enthesitis (red arrows).

A 70-year-old woman presented with fever ranging from 37,7–38,8 C, malaise, weakness and back pain lasting for 6 months. Laboratory evaluation revealed high acute phase reactants and she was treated with naproxen 500 mg twice/day, for four weeks without any significant clinical and laboratory improvement. Past medical history included hypertension treated with amlodipine, while the family history was negative. She denied headache, visual disturbances, jaw or tongue claudication and symptoms of polymyalgia rheumatica (PMR). She also denied photosensitivity, skin rashes, psoriasis, Raynaud’s phenomenon, arthritis, morning stiffness, cough, diarrhea and mucosal ulcers. Clinical examination revealed an old woman in distress. She had no signs of PMR, or arthritis no tenderness on scalp palpation and no heart and vascular murmurs. Table 1 shows details of physical examination. Laboratory tests showed low hemoglobin 10,2gr/dl, low serum ferrum 7 mg/dl (normal values > 15), and high ferritin levels 155 mg/dl (normal value < 120), a picture of anemia of chronic disease (ACD). The white blood cells and differential as well as platelets were within normal limits. The erythrocyte sedimentation rate (ESR) was 82 mm/h, the C-reactive protein (C-RP) 52 mg/l (normal value < 6), while the rest of laboratory tests including urine analysis were within normal limits. Blood and urine cultures were negative in several occasions. Antibodies for hepatitis B and C, cytomegalovirus (CMV), Epstein Barr virus (EBV) and acquired immunodeficiency virus (HIV) were all negative. Interferon gamma release assay (IGRA) was negative. Immunological tests for rheumatoid factor (RF), anticitrullinate protein antibodies (ACPA), antinuclear antibodies (ANA), antineutrophil cytoplasmic antibodies (ANCA), were negative. In addition anti-cardiolipin antibodies, serum electrophoresis and complement levels were normal. The main laboratory tests are depicted in Table 2. Chest x-rays were normal. Radiographs of the lumbar spine showed some degenerative changes at lumbar (L4-L5) and L5-Sacral (S1) vertebral levels (Fig. 1a, b) while radiographs of the pelvis were normal (Fig. 2). Computed tomography (CT) scan of lungs, upper and lower abdomen, as well as retroperitoneal space, were all unremarkable, with no sign of neoplasia. In addition, gastroscopy and colonoscopy were both normal. Heart sonography was negative for infective endocarditis. Since the radiographs of the patients showed some degenerative changes at the levels L4-L5 and L5-S1 we performed a magnetic resonance imaging (MRI) of the lumbar spine, to exclude any possibility of infectious spondylitis. However, we detected several hyper intense signals, bone marrow edema (BME), affecting many vertebral bodies from thoracic (T9) to L 2 levels, along with enthesitis (Fig. 3). These findings led us to order MRI of the pelvis which showed bilateral subchondral BME affecting both sacroiliac (SI) joints, along with enthesitis, suggesting acute sacroiliitis (Fig. 4a, b, c). In addition, HLA-B27 was positive. Thus, LO-axSpA presenting with FUO, high acute phase reactants and constitutional symptoms was diagnosed. The patient was treated with adalimumab 40 mg/ every two weeks subcutaneously with excellent response. After 2 months, the patient was free of symptoms and all laboratory tests were normalized.

**Table 1:** Clinical findings of a patient with fever of unknown origin

Clinical parameters	Clinical findings
Subjective complaints	Fever, malaise, weakness, back pain
**General clinical examination** • Blood pressure mm/Hg • Fever c° • Heart • Lungs • Peripheral vessels • Abdomen • Skin	120/80 37.9 Normal heart rate, no friction or murmur Clear, normal breath sounds bilaterally Normal pulses, no bruits Flat, without tenderness, or ascites Clear, no signs of rashes
**Examination of peripheral joints** • Shoulders • Elbows • Wrists and hands • Knees, ankles and feet	Normal range of motion, no stiffness or tenderness Normal flexion, extension, no swelling or tenderness No swelling, no tenderness Normal flexion and extension, no swelling or tenderness
**Examination of the axial skeleton** • Neck spine • Thoracic spine • Lumbar spine • Pelvis	Normal flexion, extension and rotation Normal posture, no scoliosis or kyphosis Can bend forward, below the knees. Normal Schober test No signs of hip involvement
**Examination of the muscles was normal**	Normal gait and station Intact cranial nerves Normal muscle strength of the upper and lower extremities (5/5) Normal sensory and motor examination of the peripheral nerves Normal knee and ankle reflexes

**Table 2:** Laboratory and immunological findings of a patient with fever of unknown origin

Parameters	Results	Normal range	
	At presentation	Admission	
White blood cell; (x10^9^/l)	10.150	9800	4500–11,500
Hemoglobin; g/dl	10.2	10.1	12-15.5
Platelet count; µL	350,000	355,000	150,000–420,000
Mean corpuscular volume; fL	79	78	80–98
Blood urea nitrogen; mg/dL	15	16	10–25
Creatinine; mg/dL	0.8	0.8	0.5–1.2
Calcium; mg/dL	9.3	8.2	8.5–10.1
Albumin; g/dL	4.1	4	3.4–54
Ferrum; mg/dL	7	6.5	15–31
Ferritin; ng/dL	155	156	61–120
Aspartate aminotransferase; IU/L	22	24	18–31
Alanine aminotransferase; IU/L	24	23	18–31
C-reactive protein; mg/L	52	54	0–6
Erythrocyte sedimentation rate; mm/h	82	84	0–20
Interferon-gamma release assays	Not done	Negative	-
Antinuclear antibodies titer	Negative	Negative	<’/40
Rheumatoid factor; IU/ml	Negative	Negative	0–5
Anticitrullinate protein antibodies IU/ml	Negative	Negative	0–8
Antineutrophic cytoplasmic antibodies titer	Not done	Negative	<’/20
Complement component 3; mg/dL	55	57	50–110
Complement component 4; mg/dL	15	16	10 − 50
Urinalysis	Gravity: 1024 Urine pH:5.2 No casts or red blood cells	Gravity: 1030 Urine pH: 5 No casts or red blood cells	-

### Search strategy

A search in Medline/PubMed, Scopus, Web of Science, and Directory of Open Access Journals (DOAJ) was performed on 1/7/2025 for English language sources using the following keywords: “SpA” and “fever”, “ax-SpA” and “fever”, “SpA” and “fever of unknown origin”, “ax-SpA” and “fever of unknown origin”, “late onset SpA” and “fever”, “late onset ax-SpA” and “fever of unknown origin” [7]. The results revealed no cases of LO-axSpA and FUO patients, highlighting the rarity of the specific case presentation. This case-based review follows the criteria of the CABARET recommendations of the EQUATOR network [8].

## Discussion

We described a 70-year-old woman with LO-axSpA who presented with FUO, constitutional symptoms and high acute phase reactants. Ax-SpA usually starts at the second and third decade of life. Previous studies have shown that the majority of SpA patients developed back pain before the age of 45 years [9–11]. These studies formed the basis for the Assessment of Spondyloarthritis International Society (ASAS) definition of inflammatory back pain (IBP) and the prominent place of age at onset, before the age of 45 years [12] and the subsequent development of ASAS classification criteria for ax-SpA [13]. However, several cases of late onset ankylosing spondylitis (AS) have been reported. Indeed, in a population based study in Rochester, Minnesota the incidences of AS was 7,3/100.000/years for all ages and 2.2/100.000/year after the age of 55 [14]. In another survey in Germany, only 6% of AS patients had onset of symptoms after the age of 40 [15]. The frequency of HLA-27 positivity in late onset AS was lower [9]. Late onset undifferentiated (u) SpA seems to be more common than late onset AS [2, 3]. Indeed, Dutrost and Sauvezie described 10 HLA-B27 positive men who developed oligo arthritis and peripheral pitting edema of the lower extremities, after the age of 50 years. These patients presented mild axial symptoms, constitutional symptoms and high acute phase reactants [16]. In another study, Dutrost et al. reviewed the files of seronegative rheumatoid arthritis (RA) patients after the age of 50 years. Twenty-nine out of the 105 patients, met the revised New York criteria for AS, 3 had reactive arthritis (ReA) and 44 had unclassified arthritis. Fourteen of these 44 were positive for HLA-B27, had peripheral oligo arthritis and elevated acute phase reactants, suggesting SpA [17]. Similar results were reported subsequently by Olivieri et al. who studied 23 consecutive uSpA over the age of 45 years [18, 19]. In another study a total of 157 AS patients from the Spanish registry were evaluated. Forty four (28,02%) had disease onset at age ≥50 years. The late onset group showed high frequency of neck involvement and peripheral arthritis affecting the upper and lower extremities. No differences were found concerning the genetic expression, disease activity and radiological findings [20]. Recently, age at onset of ax-SpA was studied by Boel et al. and found that around the world the great majority of ax-SpA patients (92%) had onset of axial disease, before the age of 45 years, with HLA-B27 positivity and the male gender associated with earlier disease onset [21]. It seems that onset of ax-SpA after the age of 50 is uncommon.

Fever occurs frequently in the course of many autoimmune rheumatic disease (ARD) [22–24]. However, fever has also been reported in patients with SpA [24]. On the other hand, FUO has been observed in many ARDs, but rarely in SpA patients [22–24]. Fever as an initial manifestation of SpA has been reported by Byuns et al. in a retrospective study [25]. The author described 26 patients who had fever as initial feature of SpA and compared them with 100 SpA patients, without fever as a control group. From the 26 patients, 13 (50%) had ReA, 26.9% uSpA, 15.4% AS, 3.8% PsA and 3.8% enteropathic arthritis. P-SpA was more common in febrile patients versus controls. Febrile SpA patients had less HLA-B27 positivity but had more frequently high acute phase reactants than controls [25]. In another study Guo et al. described 146 consecutive patients with enthesitis related arthritis (ERA). Among them, 52 (35.6%) had fever at disease onset, had more painful and swollen joints, and had more enthesitis. Patients with fever had higher acute phase reactants and poorer outcome, as compared with the control group [26]. Recently, Vitale et al. described 54 patients with recurrent fever episodes and no radiographic (nr)-axSpA with mean age 39, 9 ±14,8 years. All patients had fever before the SpA diagnosis as well as arthralgias (61%), myalgias (44%), arthritis (40,7%), headache (27,8%), diarrhea (25,9%), abdominal pain (24,1%) and skin reactions (22,1%). The majority of patients were treated with tumor necrosis factor inhibitors (TNFi) with an excellent response [27]. FUO in ax-SpA is extremely rare. Two case reports have been reported in patients with AS associated with aortitis [28] and enthesitis at an uncommon site [29]. More specifically, the first case was a 34year-old man with intermittent fever, weight loss, myalgia and arthralgia, he had elevated inflammatory markers and back pain. MRI of the pelvis showed findings of sacroiliitis. A positron emission tomography (PET) scan revealed inflammation of the ascending aorta, compatible with aortitis. He was treated successfully with prednisone and infliximab [28]. The other case was a 58year-old man with a history of one month fever accompanied with malaise, morning stiffness and bilateral temporal region pain. He had refractory neck and back pain since his early 20s. Radiographs of the cervical, lumbar and pelvis showed findings of SpA, which was confirmed with MRI of the sacroiliac joints. Upon further examination of temporal region pain, the MRI showed findings of enthesitis of temporal muscles. He was treated with infliximab with excellent response [29]. No patients with LO-ax SpA and FUO have been reported.

We reported an elderly woman with FUO presenting back pain, constitutional symptoms and high acute phase reactants, who was diagnosed with LO-axSpA after excluding infections, ARD, vasculitis and malignancies. The diagnosis was based on MRI of the lumbar spine findings, such as Romanus lesions, Anderson lesions and enthesitis as well as, MRI of the sacroiliac joints findings of acute sacroiliitis along with enthesitis. In addition, HLA-B27 was positive. However, the ASAS definition for sacroiliitis on MRI of sacroiliac joints is not specific, since similar MRI findings are seen in many conditions and disorders like anatomical variance, sacroilization of the fifth lumbar vertebrae, degenerative change of the lumbar spine, infections, tumors, hard workers, aging and cases mimicking ax-SpA [1, 30, 31]. Our patient except aging had no other conditions or disorders. Moreover, the patient presented many MRI lesions of the thoracic and lumbar spine suggesting SpA (Anderson and Romanus lesions, along with enthesitis) [32, 33]. On the other side, another question arises here: does the patient have a delayed diagnosis of ax-SpA [34]? Indeed, there is a gap and a delay in diagnosis of ax-SpA for approximately 6–7 years, which unfortunately did not improve the last years [35]. Our patient is a 70year-old female person, she had no family history of SpA. She never complained for symptoms of SpA and never sought consultation from general practitioners, orthopedics or rheumatologists for musculoskeletal problems, until recently, when she presented FUO along with some vague symptoms, among them back pain. We believe that our patient has a late onset ax-SpA.

LO-axSpA is underdiagnosed in the elderly population [36]. These patients deserve further attention because age population is increasing and the classification criteria for ax-SpA are not valid for elderly patients, since age at onset of ax-SpA <45 years and IBP are the major criteria [12, 13]. Thus, new classification criteria for ax-SpA including older patients are required [36]. To our knowledge, the present case is unique in the international literature, no such case has been reported so far.

In conclusion, LO-axSpA can be presented with FUO, vague constitutional symptoms and systemic inflammatory markers. In this setting, several diseases should be excluded and thorough investigation, must be performed, in order to reach the correct diagnosis.

## Data Availability

Not applicable.

## References

[1] Drosos AA, Venetsanopoulou AI, Voulgari PV (2023) Axial spondyloarthritis: evolving concepts regarding the disease’s diagnosis and treatment. Eur J Int Med 117:21–27. 10.1016/j.ejim.2023.06.026 10.1016/j.ejim.2023.06.026 37414646

[2] Toussirot E, Wendling D (2005) Late-onset ankylosing spondylitis and related spondylarthropathies: clinical and radiological characteristics and Pharmacological treatment options. Drugs Aging 22(6):451–469. 10.2165/00002512-200522060-00001 15974637 10.2165/00002512-200522060-00001

[3] Olivieri I, Pipitone N, D’ Angelo S et al (2009) Late-onset rheumatoid arthritis and late-onset spondyloarthritis. Clin Exp Rheumatol 27(4 Suppl 55):S139–S145 19822061

[4] Calin A, Elswood J, Edmunds L (1991) Late onset ankylosing spondylitis–a distinct disorder? Br J Rheumatol 30(1):69–70. 10.1093/rheumatology/30.1.69 1991225 10.1093/rheumatology/30.1.69

[5] Brophy S, Calin A (2001) Ankylosing spondylitis: interaction between genes, joints, age at onset, and disease expression. J Rheumatol 28(10):2283–2288 11669170

[6] Olivieri I, D’Angelo S, Palazzi C et al (2012) Late-onset spondyloarthritis: subset that should not be forgotten. J Rheumatol 39(6):1110–1112. 10.3899/jrheum.120184 22589252 10.3899/jrheum.120184

[7] Gasparyan AY, Ayvazyan L, Blackmore H, Kitas GD (2011) Writing a narrative biomedical review: considerations for authors, peer reviewers, and editors. Rheumatol Int 31(11):1409–1417. 10.1007/s00296-011-1999-3 21800117 10.1007/s00296-011-1999-3

[8] Benlidayi IC, Gupta L (2024) CAse-BAsed review sTandards (CABARET): considerations for Authors, Reviewers, and editors. J Korean Med Sci 39(30):e225. 10.3346/jkms.2024.39.e225 39106889 10.3346/jkms.2024.39.e225 PMC11301009

[9] Feldtkeller E, Khan MA, van der Heijde D et al (2003) Age at disease onset and diagnosis delay in HLA-B27 negative vs. positive patients with ankylosing spondylitis. Rheumatol Int 23(2):61–66. 10.1007/s00296-002-0237-4 12634937 10.1007/s00296-002-0237-4

[10] van der Linden SM, Valkenburg HA, de Jongh BM et al (1984) The risk of developing ankylosing spondylitis in HLA-B27 positive individuals. A comparison of relatives of spondylitis patients with the general population. Arthritis Rheum 27(3):241–249. 10.1002/art.1780270301 6608352 10.1002/art.1780270301

[11] Said-Nahal R, Miceli-Richard C, Berthelot JM et al (2000) The Familial form of spondylarthropathy: a clinical study of 115 multiplex families. Groupe Français d’etude Génétique des spondylarthropathies. Arthritis Rheum 43(6):1356–1365. 10.1002/1529-0131(200006)43:6%3C1356::AID-ANR20%3E3.0.CO;2-Y 10.1002/1529-0131(200006)43:6<1356::AID-ANR20>3.0.CO;2-Y 10857795

[12] Rudwaleit M, Metter A, Listing J et al (2006) Inflammatory back pain in ankylosing spondylitis: a reassessment of the clinical history for application as classification and diagnostic criteria. Arthritis Rheum 54(2):569–578. 10.1002/art.21619 16447233 10.1002/art.21619

[13] Rudwaleit M, van der Heijde D, Landewé R et al (2009) The development of assessment of spondyloarthritis international society classification criteria for axial spondyloarthritis (part II): validation and final selection. Ann Rheum Dis 68(6):777–783. 10.1136/ard.2009.108233 19297344 10.1136/ard.2009.108233

[14] Carbone LD, Cooper C, Michet CJ et al (1992) Ankylosing spondylitis in Rochester, Minnesota, 1935–1989. Is the epidemiology changing? Arthritis Rheum 35(12):1476–1482. 10.1002/art.1780351211 1472124 10.1002/art.1780351211

[15] Feldtkeller E, Bruckel J, Khan MA (2000) Scientific contributions of ankylosing spondylitis patient advocacy groups. Curr Opin Rheumatol 12(4):239–247. 10.1097/00002281-200007000-00002 10910174 10.1097/00002281-200007000-00002

[16] Dubost JJ, Sauvezie B (1989) Late onset peripheral spondyloarthropathy. J Rheumatol 16(9):1214–1217 2810278

[17] Dubost JJ, Ristori JM, Zmantar C et al (1991) [Seronegative rheumatism of late onset. Incidence and atypical forms of spondylarthropathy]. Rev Rhum Mal Osteoartic 58(9):577–584 1775904

[18] Olivieri I, Padula A, Pierro A et al (1995) Late onset undifferentiated seronegative spondyloarthropathy. J Rheumatol 22(5):899–903 8587079

[19] Olivieri I, Garcia-Porrua C, Padula A et al (2007) Late onset undifferentiated spondyloarthritis presenting with polymyalgia rheumatica features: description of seven cases. Rheumatol Int 27(10):927–933. 10.1007/s00296-007-0331-8 17426977 10.1007/s00296-007-0331-8

[20] Montilla C, Del Pino-Montes J, Collantes-Estevez E et al (2012) Clinical features of late-onset ankylosing spondylitis: comparison with early-onset disease. J Rheumatol 39(5):1008–1012. 10.3899/jrheum.111082 22422491 10.3899/jrheum.111082

[21] Boel A, López-Medina C, van der Heijde DMFM et al (2022) Age at onset in axial spondyloarthritis around the world: data from the assessment in spondyloarthritis international society peripheral involvement in spondyloarthritis study. Rheumatology (Oxford) 61(4):1468–1475. 10.1093/rheumatology/keab544 34260699 10.1093/rheumatology/keab544 PMC8996784

[22] Carsons SE (1996) Fever in rheumatic and autoimmune disease. Infect Dis Clin North Am 10(1):67–84. 10.1016/s0891-5520(05)70286-5 8698995 10.1016/s0891-5520(05)70286-5

[23] Mulders-Manders CM, Simon A, Bleeker-Rovers CP (2016) Rheumatologic diseases as the cause of fever of unknown origin. Best Pract Res Clin Rheumatol 30(5):789–801. 10.1016/j.berh.2016.10.005 27964789 10.1016/j.berh.2016.10.005

[24] Wouters JM, van de Putte LB (1986) Adult-onset still’s disease; clinical and laboratory features, treatment and progress of 45 cases. Q J Med 61(235):1055–1065 3659248

[25] Byun SJ, Bae WH, Jung SM et al (2017) Fever as an initial manifestation of spondyloarthritis: A retrospective study. PLoS ONE 12(9):e0184323. 10.1371/journal.pone.0184323 28910361 10.1371/journal.pone.0184323 PMC5598956

[26] Guo R, Cao L, Kong X et al (2015) Fever as an initial manifestation of enthesitis-related arthritis subtype of juvenile idiopathic arthritis: retrospective study. PLoS ONE 10(6):e0128979. 10.1371/journal.pone.0128979 26030261 10.1371/journal.pone.0128979 PMC4451976

[27] Vitale A, Caggiano V, Silva I et al (2023) Axial spondyloarthritis in patients with recurrent fever attacks: data from the AIDA network registry for undifferentiated autoinflammatory diseases (USAIDs). Front Med (Lausanne) 10:1195995. 10.3389/fmed.2023.1195995 37324154 10.3389/fmed.2023.1195995 PMC10263060

[28] Mehdipour Dalivand M, Abdolazimi R, Manafi-Farid R et al (2023) A case of ankylosing spondylitis presenting with fever of unknown origin diagnosed as aortitis: A case report. Clin Case Rep 11(11):e8207. 10.1002/ccr3.8207 38028057 10.1002/ccr3.8207 PMC10654463

[29] Kanda N, Takeda K, Hatakeyama S et al (2019) Ankylosing spondylitis presenting with enthesitis at an uncommon site and fever of unknown origin. BMJ Case Rep 12(8):e230113. 10.1136/bcr-2019-230113 31401572 10.1136/bcr-2019-230113 PMC6700554

[30] Renson T, de Hooge M, De Craemer AS et al (2022) Progressive increase in sacroiliac joint and spinal lesions detected on magnetic resonance imaging in healthy individuals in relation to age. Arthritis Rheumatol 74(9):1506–1514. 10.1002/art.42145 35436391 10.1002/art.42145

[31] Pelechas E, Kosta P, Voulgari PV, Drosos AA (2020) Bertolotti syndrome: a not-to-miss cause of chronic low back pain in young adults. Acta Rheumatol Port 45(1):58–60 32578578

[32] Maksymowych WP, Lambert RG, Østergaard M et al (2019) MRI lesions in the sacroiliac joints of patients with spondyloarthritis: an update of definitions and validation by the ASAS MRI working group. Ann Rheum Dis 78(11):1550–1558. 10.1136/annrheumdis-2019-215589 31422357 10.1136/annrheumdis-2019-215589

[33] Magrey MN, Danve AS, Ermann J, Walsh JA (2020) Recognizing axial spondyloarthritis: A guide for primary care. Myo Clin Proc 95(11):2499–2508. 10.1016/j.mayocp.2020.02.007 10.1016/j.mayocp.2020.02.007 32736944

[34] Hay CA, Packham J, Ryan S, Mallen CD, Chatzixenitidis A, Prior JA (2022) Diagnostic delay in axial spondyloarthritis: a systematic review. Clin Rheumatol 41(7):1939–1950. 10.1007/s10067-022-06100-7 35182270 10.1007/s10067-022-06100-7 PMC9187558

[35] Poddubnyy D, Garrido-Cumbrera M, Sommerfleck F et al (2025) Diagnostic delay in patients from the international map of axial spondyloarthritis: geographic, sociodemographic and disease-related factors. Rheumatology (Oxford) 64(4):1873–1879. 10.1093/rheumatology/keae521 39321311 10.1093/rheumatology/keae521 PMC11962975

[36] Hmamouchi I, Bahiri R, Hajjaj-Hassouni N (2011) Clinical and radiological presentations of late-onset spondyloarthritis. ISRN Rheumatol 2011:840475. 10.5402/2011/840475 23509636 10.5402/2011/840475 PMC3595659

